# Supplementary material for: Reconciling Mining with the Conservation of Cave Biodiversity: A Quantitative Baseline to Help Establish Conservation Priorities
Source: PLoS One. 2016 Dec 20;11(12):e0168348. doi: 10.1371/journal.pone.0168348 (PMC5173368; doi:10.1371/journal.pone.0168348)
Supplement: S1 Dataset — (ZIP) [file pone.0168348.s002.zip › Taxa/Serra Norte/SN_2013/N4E/Lista N4E-21.pdf]

CAVIDADE N4E-0021

Classe	Ordem	Fam/Outros	Gên/Outros	Espécie	S-U	U-S	Zon.
Annelida	Oligochaeta			jovem		2	E/P
Arachnida	Acari	(Oribatida)		jovem	X		
Arachnida	Acari	(Parasitengonina)		sp.13		X	P
Arachnida	Acari	Argasidae	*Ornithodoros*	sp.1		X	P
Arachnida	Acari	Eupodidae	*Linopodes*	sp.	X	X	E
Arachnida	Acari	Mesostigmata		jovem	X		
Arachnida	Acari	Ologamasidae		sp.	X		
Arachnida	Acari	Trombiculidae		sp.39	X	X	E
Arachnida	Amblypygi	Charinidae	*Charinus*	sp.1	X		
Arachnida	Amblypygi	Phrynidae	*Heterophrynus*	*longicornis*	X	3	
Arachnida	Araneae	Araneidae	*Alpaida*	*smila*		X	E
Arachnida	Araneae	Corinnidae		jovem		1	E
Arachnida	Araneae	Ctenidae		jovem	1		
Arachnida	Araneae	Nesticidae	*Nesticus*	sp.2		X	E/P
Arachnida	Araneae	Ochyroceratidae	*Ochyrocera*	sp.1	X	X	E/P
Arachnida	Araneae	Ochyroceratidae	*Speocera*	sp.1		X	E/P
Arachnida	Araneae	Pholcidae	Ninetinae	sp.1		X	E
Arachnida	Araneae	Segestriidae		jovem		X	E
Arachnida	Araneae	Theridiidae		jovem	X		
Arachnida	Araneae	Theridiosomatidae	*Plato*	sp.1	X	X	E/P
Arachnida	Opiliones	Escadabiidae		sp.1	X		
Arachnida	Opiliones	Escadabiidae		sp.2	X	X	E/P
Arachnida	Palpigradi	Eukoeneniidae	*Allokoenenia*	sp.1		X	E/P
Arachnida	Palpigradi	Eukoeneniidae	*Eukoenenia*	sp.1		X	E
Arachnida	Schizomida	Hubbardiidae	aff. *Rowlandius*	sp.1		X	E
Chilopoda	Scolopendromorpha	Scolopocryptopidae	*Dinocryptops*	*miersii*	X		
Diplopoda	Polydesmida	Chelodesmidae		sp.3		1	P
Diplopoda	Polydesmida	Pyrgodesmidae		sp.1	X	X	E/P
Entognatha	Collembola	Cyphoderidae		sp.1		X	E
Entognatha	Collembola	Isotomidae		sp.1		X	E
Entognatha	Collembola	Paronellidae		sp.1		X	E
Entognatha	Collembola	Paronellidae		sp.4		X	E/P
Entognatha	Collembola	Sminthuroidea		sp.2	X	X	E/P
Entognatha	Diplura	Campodeidae		sp.1	X	X	E
Insecta	Blattodea	Blattidae		jovem	X	1	E
Insecta	Coleoptera	Carabidae	*Coarazuphium*	sp.n.1		X	P
Insecta	Coleoptera	Carabidae	Gen.n.	sp.17		X	P
Insecta	Coleoptera	Carabidae		sp.3	X	X	E
Insecta	Coleoptera	Staphylinidae	Pselaphinae	sp.12		X	E
Insecta	Coleoptera	Staphylinidae	Pselaphinae	sp.35	X	X	E
Insecta	Diptera	Ceratopogonidae		sp.	X		
Insecta	Diptera	Chironomidae		sp.	X		
Insecta	Diptera	Clusiidae		sp.	X		
Insecta	Diptera	Dolichopodidae		sp.		X	E/P
Insecta	Diptera	Drosophilidae	*Drosophila*	sp.	X		
Insecta	Diptera	Mycetophilidae		sp.	X		
Insecta	Diptera	Psychodidae	*Sciopemyia*	*sordellii*	X	X	E
Insecta	Heteroptera	Cydnidae		sp.	X	X	P
Insecta	Heteroptera	Dipsocoroidea		sp.		X	E
Insecta	Homoptera	Cixiidae		sp.	X	X	E
Insecta	Hymenoptera	Formicidae	*Apterostigma*	sp.	X		
Insecta	Hymenoptera	Formicidae	*Brachymyrmex*	sp.1		X	E
Insecta	Hymenoptera	Formicidae	*Camponotus*	*atriceps*	X		
Insecta	Hymenoptera	Formicidae	*Hypoponera*	sp.1		X	P
Insecta	Hymenoptera	Formicidae	*Pachycondyla*	sp.3		X	E

Insecta	Hymenoptera	Formicidae	*Pachycondyla*	*striata*	X		
Insecta	Hymenoptera	Formicidae	*Paratrechina*	sp.	X		
Insecta	Hymenoptera	Formicidae	*Pheidole*	sp.	X		
Insecta	Hymenoptera	Formicidae	*Solenopsis*	sp.1	X	X	E
Insecta	Lepidoptera	Tineoidea		sp.	X	X	
Insecta	Orthoptera	Phalangopsidae	*Phalangopsis*	sp.	X	70	E
Insecta	Psocoptera			jovem	X	X	E
Insecta	Thysanura	Nicoletiidae	Nicoletiinae	sp.1	X		
Nematoda				sp.1		X	E
Mammalia	Chiroptera	Phyllostomidae	*Carollia*	*perspicillata*		3	P/A
Mammalia	Chiroptera	Phyllostomidae	*Glossophaga*	*soricina*		6	P/A
Mammalia	Chiroptera			sp.	50		E/P/A
